# Winding loss analysis of planar spiral coil and its structure optimization technique in wireless power transfer system

**DOI:** 10.1038/s41598-022-24006-x

**Published:** 2022-11-12

**Authors:** Qingbin Chen, Xu Zhang, Wei Chen, Cong Wang

**Affiliations:** 1 College of Electrical Engineering and Automation, Fuzhou University, Fuzhou, 350108 China; 2 Maintenance Company of State Grid Jiangxi Electric Power Co., LTD, Nanchang, China

**Keywords:** Electrical and electronic engineering, Energy infrastructure

## Abstract

Circular planar spiral coils are wildly used for the magnetic coupling system in a high-frequency wireless power transfer system. The loss of the magnetic coupling system usually takes dominance in the whole system. This paper built the calculation model of magnetic field strength and coil loss, the proposed calculation model can effectively consider the mutual influence between the transmitter and receiver coil and accurately calculated the AC loss of WPT coils. Then, the effect of turn spacing on the AC resistance of coil is analyzed. It reveals that the proximity effect loss is greater when the coil is tightly wound, and the AC loss can be optimized by designing the turn spacing. Based on the above analysis, a double-layer coil method is proposed. This method can reduce the AC loss and improve the quality factor (*Q*) without changing the mutual inductance and footprint of coil at high frequency. The AC resistance of the double-layer coil method can be greatly reduced compared with the general method through simulation and experiment. The work efficiency of WPT system is increased by 4.3%, which verifies the accuracy and flexibility of theoretical analysis.

## Introduction

Wireless power transfer (WPT) technology has been widely investigated and applied in some applications, such as implantable medical devices, electronic products, portable devices, etc. It can avoid the disadvantages of using wires and sockets^1–5^.

The magnetic resonant WPT technology is the most widely studied and applied because of its transfer distance and power grade^6–8^. The characteristics of the magnetic coupling systems (MCS), including gain, transfer distance, efficiency, and stability, can affect the operating performance of the whole WPT system. And its loss occupies 30% to 50% of the WPT system loss^9–11^. Thus, its optimization technique is extremely important to improve the efficiency of WPT system. The magnetic core is not usually used in long transfer distance and high-frequency applications. As a result, the air planar spiral coil optimization becomes increasingly important in the WPT MCS’s design.

For the turn spacing optimization of the solid coil, the current method mainly aims to improve the *Q* value. In^12^, the influence of coil turns and turn spacing on self-inductance and AC resistance is analyzed. An optimal design of limited-size wireless power transmission coil is proposed. The optimal *Q* value is obtained by changing the turns and turn spacing in the case of constant coil size. In^13^, a method for optimizing the ratio of mutual inductance *M* and the AC resistance of coils *R*_ac_ is proposed. This method can obtain the maximum value *M*/*R*_ac_ by designing the turns and turn spacing. However, keeping the coil inductance, footprint, and turns constant is difficult in these methods.

The variable winding width scheme and multiple-layer structure coil have been widely studied to optimize printed circuit board (PCB) coil. In^14,15^, the variable winding width scheme is proposed to reduce the high-frequency AC loss. However, changing the width of each turn for a solid wire coil is impractical. The connection of adjacent turns requires welding, and the welding resistance between adjacent turns cannot be ignored. Double-layer and multilayer coils have been designed for medical implants^16,17^. They can provide a high-power efficiency in a limited space by stacking layers of coils together rather than enlarging the coil diameter. However, the coil is a PCB structure, and the traditional AC resistance calculation model of PCB coil is one dimension and unsuitable for double-layer and multilayer coils. The loss of double-layer coil in current schemes usually be obtained by simulation. At the same time, the current double-layer coil structure mainly improves *Q* by increasing inductance instead of reducing the AC resistance of the coil. In^18^, a double spiral coil is proposed, and it can improve the coupling coefficient of the WPT system by increasing the interlayer distance. When the interlayer distance is large enough, the quality factor can be improved. However, the too large interlayer distance may lead to an increase in coil volume.

To reduce the skin effect loss of MCS coil, the Litz wire is usually used to improve the efficiency of the WPT system instead of solid coil^19^. However, Litz wire has many stranded wires and thin strands, which results in the proximity effect loss of Litz wire being larger than the solid wire in high-frequency applications^20,21^. If the frequency exceeds several hundred kHz, the proximity effect will be greatly enhanced and the coil loss will greatly increase.

The current is distributed to the conductor surface at high frequency due to the interaction of magnetic field between two adjacent conductors, thereby increasing the proximity effect loss. The turn spacing of a tightly wound coil is extremely small, resulting in a greater proximity effect and AC resistance to the coil. Thus, the efficiency of WPT system can be improved by designing the turn spacing of MCS coil.

This paper analyzes the AC resistance of the single-turn coil, then deduces the planar spiral coil's AC resistance model and proposes the coil's AC resistance model, which can take the mutual influence between the transmitter and the receiver coil into consideration. Based on the model, a double-layer coil structure is proposed to reduce the winding loss of MCS and improve the WPT system's efficiency without changing the mutual inductance and footprint of coil. Finally, it verifies the correctness of the theoretical analysis by comparing the simulation and experimental results.

The rest of this paper is organized as follows. "Loss analysis of single-turn planar spiral coil" section 1.1 builds the magnetic field strength and the loss calculation model of the air planar spiral coil, and analyzes the effect of turn spacing on the AC resistance. Section 3 proposes the double-layer coil structure. Section 4 analyzes the optimized coil by simulation and experimental, and compares the related schemes.

### Loss analysis of single-turn planar spiral coil

**Figure 1:**
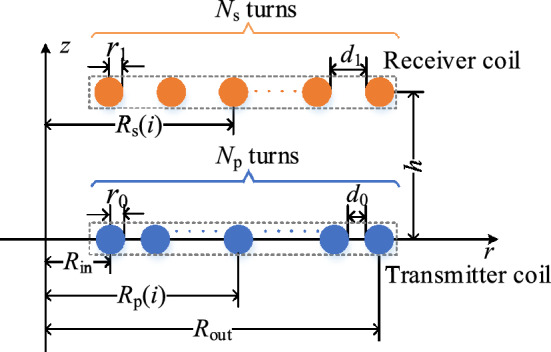
The 2D model of planar spiral coil in finite element analysis (FEA) simulation.

Each turn of the air planar spiral coil in the WPT system can be simplified as a concentric circle coil, and its two-dimensional (2D) model in the *r*-*z* coordinate system is shown in Fig. 1.

The size of the MCS includes inner diameter *R*_in_, outer diameter *R*_out_, and transmission distance *h*. And the transmitter coil parameters include wire gauge 2*r*_0_, turn spacing *d*_0_, the radius of the concentric circle of the *i*-th turn *R*_p_(*i*) and turns *N*_p_, respectively. Moreover, the receiver coil parameters include wire gauge 2*r*_1_, turn spacing *d*_1_, the radius of the concentric circle of the *i*-th turn *R*_s_(*i*) and turns *N*_s_, respectively.

### Loss model of the single-turn coil

**Figure 2:**
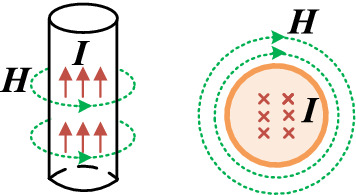
The distribution of ***H*** and ***I*** in single-turn coil.

In the WPT system, the coil loss is related to current ***I*** in the coil and the external magnetic field strength ***H*** in Fig. 2.

**Figure 3:**
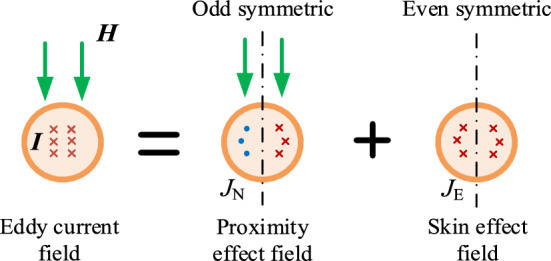
2D model of eddy current field.

Firstly, this paper analyzed the loss model of single-turn air planar spiral coil. Furthermore, the high frequency eddy current model of single-turn planar spiral coil is shown in Fig. 3. According to the high frequency eddy current loss mechanism, the AC loss of coil includes skin effect loss and proximity effect loss, which are orthogonal. The current ***I*** in the coil will result in the skin effect loss, and the external magnetic field ***H*** of the coil will result in the proximity effect loss.

In Fig. 3, · represents the direction of ***I*** from the inside to the outside of the screen, and × represents the oppsite direction compared to ·.

**Figure 4:**
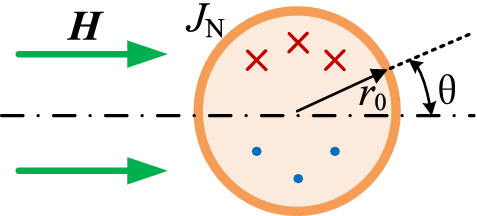
The distribution of the proximity current density.

According to Bessel and Kelvin functions, the skin current density *J*_E_(*r*_0_) and the proximity current density *J*_N_(*r*_0_) can be calculated in Eq. (1). And the current density of proximity effect is shown in Fig. 4. It can be seen from Eq. (1) that *J*_E_(*r*_0_) is only related to current ***I*** and *J*_N_(*r*_0_) is only related to the external magnetic field ***H***.

1 $$ \left\{ {\begin{array}{*{20}l} {J_{E} (R(i)) = \frac{{j^{3/2} \xi \cdot \left| I \right|}}{{2\pi r_{0} }} \cdot \frac{{J_{0} (j^{3/2} \xi R(i))}}{{J_{1} (j^{3/2} \xi \cdot r_{0} )}}} \hfill \\ {J_{N} (R(i),\theta ) = 2j^{3/2} \xi \cdot \frac{{J_{1} (j^{3/2} \xi R(i))}}{{J_{0} (j^{3/2} \xi r_{0} )}}\left| H \right|\sin \theta } \hfill \\ {\Gamma_{{{\text{skin}}}} = \frac{{ber(\xi ,r_{0} ) \cdot bei^{\prime}(\xi ,r_{0} ) - ber^{\prime}(\xi ,r_{0} ) \cdot bei(\xi ,r_{0} )}}{{ber^{{\prime}{2}} (\xi ,r_{0} ) + bei^{{\prime}{2}} (\xi ,r_{0} )}}} \hfill \\ {\Gamma_{{{\text{pro}}}} = \frac{{ber_{2} (\xi ,r_{0} ) \cdot ber^{\prime}(\xi ,r_{0} ) - bei_{2} (\xi ,r_{0} ) \cdot bei^{\prime}(\xi ,r_{0} )}}{{ber^{2} (\xi ,r_{0} ) + bei^{2} (\xi ,r_{0} )}}} \hfill \\ {\xi = \sqrt {2\pi f_{s} \cdot \mu_{0} \cdot \sigma } = \frac{\sqrt 2 }{\delta }} \hfill \\ \end{array} ,} \right. $$

where *μ*_0_ and *σ* are the magnetic permeability and the conductivity of the conductor, respectively. *f*_s_ is the operation frequency, *δ* is the skin depth and *R*(*i*) is the radius of *i*-th turn coil. *ber* and *bei* are Kelvin functions, while *J*_1_ and *J*_0_ are Bessel functions.

Then, combined with the superposition theorem, the AC loss of single-turn coil *P*_turn_ can be obtained by Eq. (2).

2 $$  \left\{ {\begin{array}{*{20}l}    {P_{{{\text{turn}}}}  = 2\pi R(i) \cdot (P_{{\text{E}}}  + P_{{\text{N}}} )} \hfill  \\    {P_{{\text{E}}}  = \frac{{\xi  \cdot \left| \user2{I} \right|^{2}  \cdot \Gamma _{{{\text{skin}}}} }}{{2\pi  \cdot r_{0}  \cdot \sigma }}} \hfill  \\    {P_{{\text{N}}}  = \frac{{ - 2\pi  \cdot r_{0}  \cdot \xi  \cdot \left| \user2{H} \right|^{2}  \cdot \Gamma _{{{\text{pro}}}} }}{\sigma }} \hfill  \\   \end{array} ,} \right. $$

*P*_E_ is the skin effect loss, and *P*_N_ is the proximity effect loss, where *P*_E_ and *P*_N_ are linear functions with |***I***|^2^.

The AC resistance of the single-turn coil is calculated by Eq. (3).

3 $$  R_{{{\text{ac}}}}  = \frac{{2P_{{{\text{turn}}}} }}{{\left| \user2{I} \right|^{2} }},  $$

**Figure 5:**
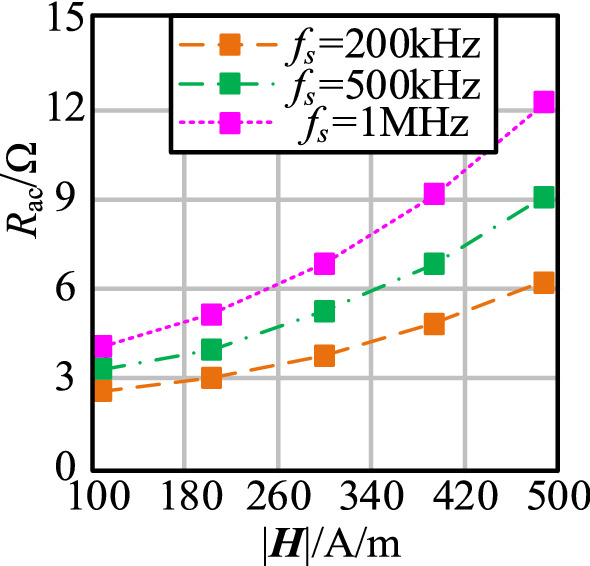
The change curve of *R*_ac_ with |***H***| at different *f*_s_.

In accordance with Eq. (3), *R*_ac_ varies with magnetic field strength ***H*** in different operation frequencies *f*_s_ when wire gauge 2*r*_0_ = 1.12 mm, as shown in Fig. 5.

As shown in Fig. 5, the AC resistance of the coil increases with the increase in magnetic field strength |***H***|. *R*_ac_ is larger in high-operation frequency.

However, the external magnetic field strength ***H***, which results in the proximity effect loss in the WPT system, is affected by other coil turns. Thus, the calculation model of magnetic field strength ***H*** of the whole coil needs to be established.

### Loss model of more-turn planar spiral coil

**Figure 6:**
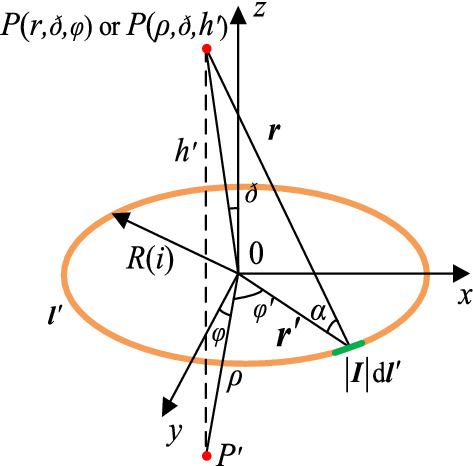
Three-dimensional model of single-turn coil in rectangular coordinate system.

Taking the transmitter coil as an example, the 3D model of the single-turn WPT coil is shown in Fig. 6. The magnetic field strength can be calculated by the magnetic vector, where *P'* is the projection of *P* on the *xy* plane, *h'* is the distance between *P* and *P'*, and *ρ* is the distance between *P'* and point 0.

When the turn spacing is large, the working current ***I*** is concentrated in the center of the conductor. The magnetic vector ***A*** of point *P* generated by the single-turn coil can be expressed as Eq. (4).

4 $$  \left\{ {\begin{array}{*{20}l}    {{A} = \frac{\mu }{{4\pi }}\oint\limits_{{2l^{{\prime }} }} {\frac{{\left| {I} \right|d{l}^{{\prime }} }}{{\left| {{r} - {r}^{{\prime }} } \right|}}} {\text{ }}} \hfill  \\    {\left| {I} \right|d{l}^{{\prime }}  = \left| {I} \right| \cdot R\left( i \right) \cdot \left( { - \sin \varphi ^{{\prime }} e_{x}  + \cos \varphi ^{{\prime }} e_{y} } \right)d\varphi ^{{\prime }} } \hfill  \\    {\left| {{r} - {r}^{{\prime }} } \right| = \sqrt {R\left( i \right)^{2}  + r^{2}  - 2R\left( i \right) \cdot r \cdot \cos \alpha } } \hfill  \\   \end{array} } \right.,  $$

Then, the magnetic field strength ***H*** at point *P* can be calculated by ${\varvec{H}} = {{\nabla \times {\varvec{A}}} \mathord{\left/ {\vphantom {{\nabla \times {\varvec{A}}} {\mu_{0} }}} \right. \kern-\nulldelimiterspace} {\mu_{0} }}$. In a cylindrical coordinate system, the magnetic field strength has ***e***_ρ_ component and ***e***_z_ component because ***A*** has only ***φ*** component. The ***e***_ρ_ and ***e***_z_ components of magnetic field strength are expressed as:

5 $$ \left\{ {\begin{array}{*{20}l} {\left| {{\varvec{H}}_{{{\varvec{\uprho}}}} {\mathbf{(}}{\varvec{R}}{(}{\varvec{i}}{),}{\varvec{\rho}}{,}\user2{h^{\prime}}{\mathbf{)}}} \right| = \frac{{\left| {\varvec{I}} \right|\user2{h^{\prime}} \cdot [\frac{{A(k)(\rho^{2} + R(i)^{2} + \user2{h^{\prime}}^{2} )}}{{(\rho - R(i))^{2} + \user2{h^{\prime}}^{2} }} - F(k)]}}{{2\pi \rho \sqrt {(\rho + R(i))^{2} + \user2{h^{\prime}}^{2} } }}} \hfill \\ {\left| {{\varvec{H}}_{{\mathbf{z}}} {\mathbf{(}}{\varvec{R}}{(}{\varvec{i}}{),}{\varvec{\rho}}{,}\user2{h^{\prime}}{\mathbf{)}}} \right| = \frac{{\left| {\varvec{I}} \right| \cdot [\frac{{A(k)(R(i)^{2} - \rho^{2} - \user2{h^{\prime}}^{2} )}}{{(\rho - R(i))^{2} + \user2{h^{\prime}}^{2} }} + F(k)]}}{{2\pi \sqrt {(\rho + R(i))^{2} + \user2{h^{\prime}}^{2} } }}} \hfill \\ {k(R(i),\rho ,\user2{h^{\prime}}) = \frac{4R(i)\rho }{{\left( {R(i) + \rho } \right)^{2} + \user2{h^{\prime}}^{2} }}} \hfill \\ {F(k) = \int_{0}^{\pi /2} {\frac{1}{{\sqrt {1 - k(R(i),\rho ,\user2{h^{\prime}})\sin^{2} (\alpha )} }}} {d}\alpha } \hfill \\ {A(k) = \int_{0}^{\pi /2} {\sqrt {1 - k(R(i),\rho ,\user2{h^{\prime}})\sin^{2} (\alpha )} } {d}\alpha } \hfill \\ \end{array} } \right., $$

In accordance with the model of WPT coil in Fig. 1, the mutual influence of the primary and secondary windings is considered when solving the magnetic field strength in coil. Take the *i*-th turn transmitter coil as an example, the magnetic field strength generated by the other (*N*_p_ − 1) turns transmitter coils has only the ***e***_z_ component ***H***_**pz**_(*i*), which can be expressed as:

6 $$  \left| {\user2{H}_{{{\text{pz}}}} (i)} \right| = \sum\limits_{{j = 1 \wedge j \ne i}}^{{N_{p} }} {\frac{{\left| \user2{I} \right| \cdot \left[ {\frac{{R_{{\text{p}}} \left( j \right)^{2}  - R_{{\text{p}}} \left( i \right)^{2} }}{{\left( {R_{{\text{p}}} \left( i \right) - R_{{\text{p}}} \left( j \right)} \right)^{2} }}A(k) + F(k)} \right]}}{{2\pi \sqrt {\left( {R_{{\text{p}}} \left( j \right) + R_{{\text{p}}} \left( i \right)} \right)^{2} } }},}   $$

The magnetic field strength generated by the *N*_s_ turns receiver coils has the ***e***_z_ component ***H***_**sz**_(*i*) and the ***e***_ρ_ component ***H***_**sρ**_(*i*), which can be expressed as:

7 $$  \left\{ {\begin{array}{*{20}l}    {\left| {\user2{H}_{{{\text{sz}}}} (i)} \right| =  - \sum\limits_{{j = 1}}^{m} {\frac{{\left| \user2{I} \right| \cdot \left[ {\frac{{R_{{\text{s}}} \left( j \right)^{2}  - R_{{\text{p}}} \left( i \right)^{2}  - h^{2} }}{{\left( {R_{{\text{s}}} \left( j \right) - R_{{\text{p}}} \left( i \right)} \right)^{2}  + h^{2} }}A(k) + F(k)} \right]}}{{2\pi \sqrt {\left( {R_{{\text{s}}} \left( j \right) + R_{{\text{p}}} \left( i \right)} \right)^{2}  + h^{2} } }}}  + \sum\limits_{{j = m + 1}}^{{N_{s} }} {\frac{{\left| \user2{I} \right| \cdot \left[ {\frac{{R_{{\text{s}}} \left( j \right)^{2}  - R_{{\text{p}}} \left( i \right)^{2}  - h^{2} }}{{\left( {R_{{\text{s}}} \left( j \right) - R_{{\text{p}}} \left( i \right)} \right)^{2}  + h^{2} }}A(k) + F(k)} \right]}}{{2\pi \sqrt {\left( {R_{{\text{s}}} \left( j \right) + R_{{\text{p}}} \left( i \right)} \right)^{2}  + h^{2} } }}} } \hfill  \\    {\left| {\user2{H}_{{{\text{sp}}}} (i)} \right| = \sum\limits_{{j = 1}}^{{N_{s} }} {\frac{{\left| \user2{I} \right|h \cdot \left[ {\frac{{R_{{\text{s}}} \left( j \right)^{2}  - R_{{\text{p}}} \left( i \right)^{2}  - h^{2} }}{{\left( {R_{{\text{s}}} \left( j \right) - R_{{\text{p}}} \left( i \right)} \right)^{2}  + h^{2} }}A(k) + F(k)} \right]}}{{2\pi \sqrt {\left( {R_{{\text{s}}} \left( j \right) + R_{{\text{p}}} \left( i \right)} \right)^{2}  + h^{2} } }}} } \hfill  \\   \end{array} } \right.,   $$

where *m* is obtained by *R*_s_(*m*) ≤ *R*_p_(*i*) < *R*_s_(*m* + 1).

When the turn spacing is small, the proximity effect is larger, resulting in the uneven current distribution in the coil. An additional magnetic field strength ***H***_**add**_ is found.

**Figure 7:**
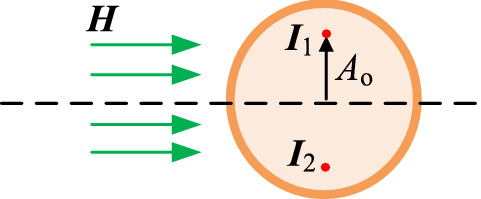
Equivalent point current sources of proximity effect field.

*J*_N_(*r*_0_) is equivalent to two current sources ***I***_**1**_ and ***I***_**2**_ with the same value and opposite direction in solving ***H***_**add**_. The direction of ***I***_**1**_ is from the outside to the inside of the screen, while the direction of ***I***_**2**_ is opposite to ***I***_**1**_, as shown in Fig. 7.

where *A*_o_ is the distance between the equivalent current sources and the center of the conductor. It can be solved using Eqs. (8) and (9) based on the uniqueness theorem.

8 $$  \left\{ \begin{gathered}   A_{o}  = c \cdot r_{0}  \hfill \\   \left| {\user2{I}_{{{\text{add}}}} (c)} \right| = \frac{{\left| \user2{H} \right|}}{2} \hfill \\   \left| {\user2{I}_{{{\text{add}}}} (c)} \right| = \int_{0}^{\pi } {\int_{0}^{{r_{0} }} {J_{{\text{N}}} (R(i),\theta ,c)r{\text{d}}r{\text{d}}\theta } }  \hfill \\  \end{gathered}  \right.,  $$

9 $$  J_{{\text{N}}} \left( {R(i),\theta ,c} \right) = \left\{ {\begin{array}{*{20}l}    {J_{{\text{N}}} \left( {R(i),\theta ,c} \right),} \hfill & {cr_{0}  \ge R(i){\text{sin}}\theta } \hfill  \\    {0,} \hfill & {{\text{other}}} \hfill  \\   \end{array} } \right.,  $$

The value of current sources and their location can be obtained in accordance with Eqs. (8) and (9). Thus, ***H***_**add**_(*i*) in the *i*-th turn coil can be expressed as Eq. (10).

10 $$  \left\{ {\begin{array}{*{20}l}    {\left| {\user2{H}_{{{\text{add}}}} (i)} \right| = \sum\limits_{{j = 1 \wedge j \ne i}}^{N} {\user2{H}_{{{\text{a}}\rho }} (j) \cdot e_{\rho }  + \user2{H}_{{{\text{az}}}} (j) \cdot e_{{\text{z}}} } } \hfill  \\    {\user2{H}_{{{\text{a}}\rho }} (j) = H_{\rho } \left( {R(i) - A_{{{\text{o}}\rho }} ,R(j),h + A_{{{\text{oz}}}} ,\left| {\user2{I}_{{{\text{add}}}} } \right|} \right)} \hfill  \\    {\user2{H}_{{{\text{az}}}} (j) = \user2{H}_{{\text{z}}} \left( {R(i) + A_{{{\text{o}}\rho }} ,R(j),h - A_{{{\text{oz}}}} ,\left| {\user2{I}_{{{\text{add}}}} } \right|} \right)} \hfill  \\   \end{array} } \right.,  $$

where *N* is the coil turns, and *A*_oρ_ and *A*_oz_ are the ***e***_ρ_ component and ***e***_z_ component of *A*_o_, respectively.

**Figure 8:**
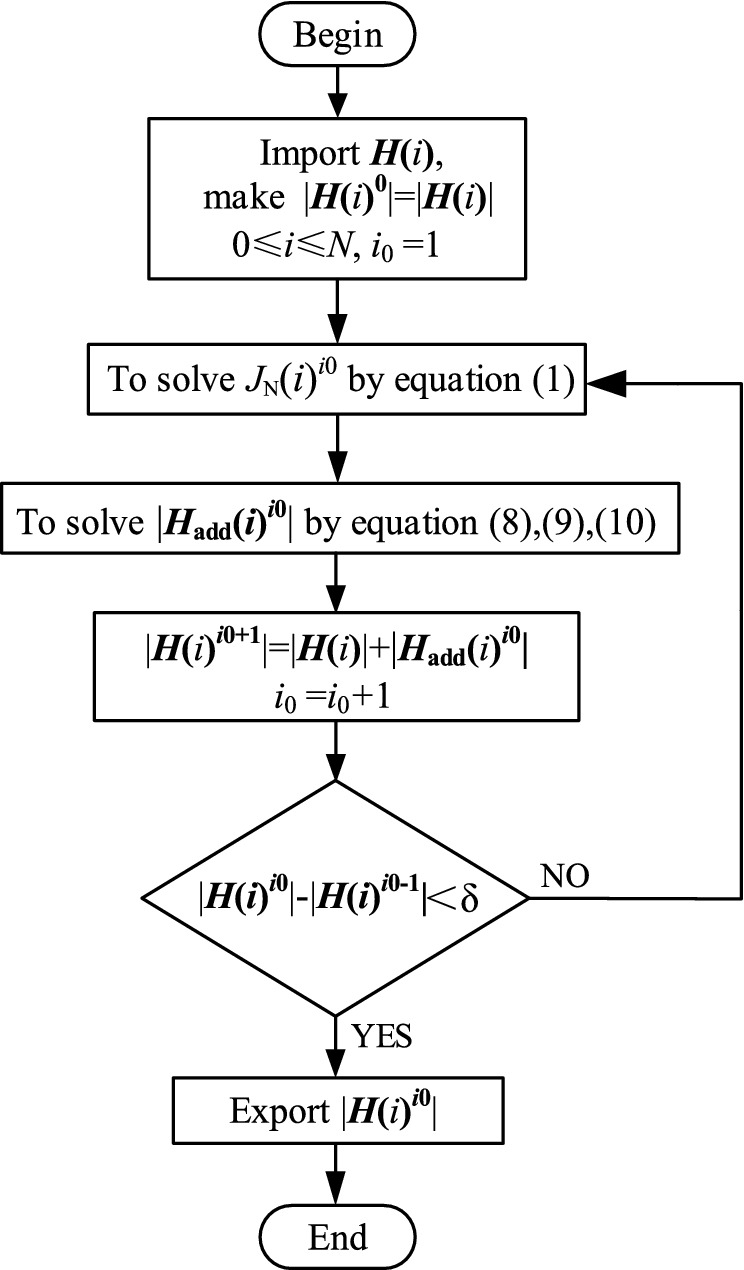
Calculation process of |***H***(*i*)|.

The magnetic field strength ***H***(*i*) in the *i*-th turn coil can be solved using the process in Fig. 8.

*i*_0_ is the number of iterations, and *δ* is the maximum error of |***H***(*i*)|.

For the solution of |***H***(*i*)| of the model in Fig. 1, |***H***_**pz**_(*i*)|, |***H***_**sz**_(*i*)|, and |***H***_**sρ**_(*i*)| can be further improved by using the process in Fig. 8, and the final |***H***_**pz**_(*i*)^***i*0**^|, |***H***_**sz**_(*i*)^***i*0**^|, and |***H***_**sρ**_(*i*)^***i*0**^| can be obtained in accordance with the superposition theorem:

11 $$ \left| {{\varvec{H}}{\mathbf{(}}i{\mathbf{)}}} \right| = \sqrt {(\left| {{\varvec{H}}_{{{\mathbf{pz}}}} {\mathbf{(}}i{\mathbf{)}}^{{{\varvec{i}}{\mathbf{0}}}} } \right| + \left| {{\varvec{H}}_{{{\mathbf{sz}}}} {\mathbf{(}}i{\mathbf{)}}^{{{\varvec{i}}{\mathbf{0}}}} } \right|)^{2} + \left| {{\varvec{H}}_{{{\mathbf{s\rho }}}} {\mathbf{(}}i{\mathbf{)}}^{{{\varvec{i}}{\mathbf{0}}}} } \right|^{2} } , $$

**Figure 9:**
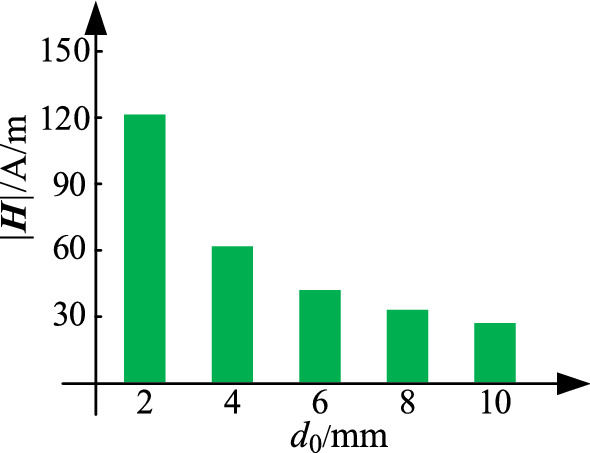
Curve of *|****H****|* under different turn spacing *d*_0_.

For a two-turn coil with a fixed wire gauge (2*r*_0_ = 1.2 mm), the turn spacing will change by changing the radius of the coil outside along the *x*-axis when the radius of the coil inside is determined (e.g., *R* = 0.1 m). This condition results in different magnetic field strengths in the coil inside, as shown in Fig. 9.

As shown in Fig. 9, the magnetic field strength in the coil decreases with the increase in the turn spacing. The influence of the proximity effect is weakened, which may decrease the AC resistance of the coil. Thus, *R*_ac_ can be reduced by increasing the turn spacing. In the actual application, the turn spacing can be changed by increasing the distance on the *z*-axis. Compared with the radius of the coil turns, the increase in the coil thickness can be ignored.

### Double-layer coil scheme

Air planar spiral coil with a one-layer structure is usually adopted, and its thickness is mainly determined by wire gauge, which can be ignored compared with its diameter. Therefore, the moving part of the coil along the *z*-axis direction at the distance of the wire gauge size will increase the coil's turn spacing and affect the coil's proximity effect but not the coil's volume. By moving the coil turns, the single-layer coil becomes a multilayer coil, which greatly increases the turn spacing and reduces the AC resistance. However, it will increase the interlayer capacitance of the coil. As the number of coil layers increases, the interlayer capacitance of the coil will greatly increase, reducing the coil's high-frequency characteristics. Therefore, after comprehensively considering the value of coil AC resistance, coil's space volume and interlayer capacitance, this paper selected a double-layer coil as the optimization scheme.

A double-layer coil structure is proposed. In this method, the turn spacing is increased by moving coils along the *z*-axis direction to keep the coil's inner diameter and outer diameter, turns, and wire gauge constant. Taking the transmitter coil for an example, an interlaced double-layer and parallel double-layer coil is designed in this study.

The moving distance should not be extremely large. The moving distance *Z*_0_ is selected as two multiples of *r*_0_ for the convenience of comparison and making of coils in this study. The optimization effect is noticeable, and the thickness of the double-layer coil is negligible compared with its width. *Z*_0_ can be chosen as any value for keeping the coil volume unchanged and is not restricted by the wire gauge.

**Figure 10:**
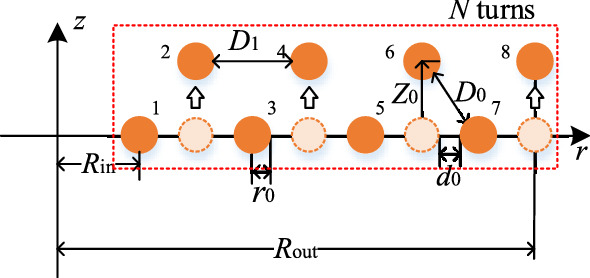
Two-dimensional model of interlaced double-layer coil.
  The moving distance of coil *Z*_0_ is several times larger than *r*_0_ in this study. Compared with the model of the coil in Fig. 1, the turn spacing *d*_0_ changes to *D*_0_ and *D*_1_ in the interlaced double-layer coil, which can be expressed as:
  12 $$ \left\{ {\begin{array}{*{20}l} {D_{0} = \sqrt {(d_{0} + 2r_{0} )^{2} + Z_{0}^{2} } - 2r_{0} } \hfill \\ {D_{1} = 2d_{0} + 2r_{0} } \hfill \\ \end{array} } \right., $$
- Parallel double-layer coil.
  The 2D model of the parallel double-layer coil is shown in Fig. 11.

- Interlaced double-layer coil.
  The 2D model of the interlaced double-layer coil is shown in Fig. 10.

**Figure 11:**
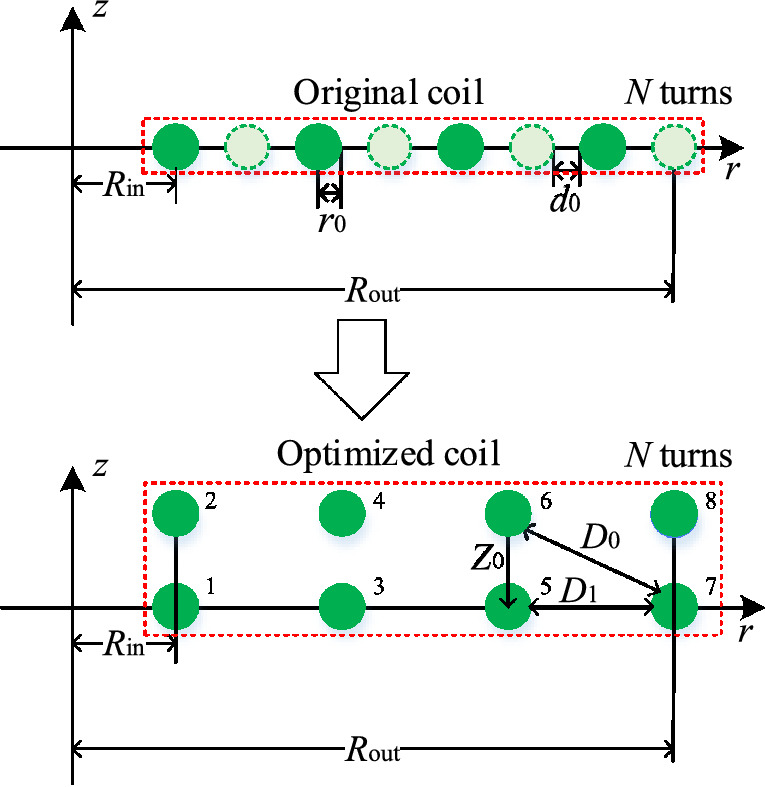
Two-dimensional model of parallel double-layer coil.
  In the parallel double-layer coil, the turn spacing is *Z*_0_, and *D*_0_ and *D*_1_ can be expressed as:
  13 $$  \left\{ \begin{gathered}   d_{0}  = \frac{{R_{{{\text{out}}}}  - R_{{{\text{in}}}} }}{{N - 1}} - 2r_{0}  \hfill \\   D_{0}  = \sqrt {\left( {D_{1}  + 2r_{0} } \right)^{2}  + Z_{0} ^{2} }  - 2r_{0}  \hfill \\  \end{gathered}  \right.,  $$
  14 $$  D_{1}  = \left\{ {\begin{array}{*{20}l}    {\frac{{R_{{{\text{out}}}}  - R_{{{\text{in}}}} }}{{{N \mathord{\left/ {\vphantom {N 2}} \right. \kern-\nulldelimiterspace} 2} - 1}} - 2r_{0} ,} \hfill & {N\;is\;{\text{even}}} \hfill  \\    {\frac{{2\left( {R_{{{\text{out}}}}  - R_{{{\text{in}}}} } \right)}}{{N - 1}} - 2r_{0} ,} \hfill & {N\;{\text{is}}\;{\text{odd}}} \hfill  \\   \end{array} } \right.,  $$
  For the solution of AC resistance in the double-layer coil, each coil turn can be simplified as a concentric circle coil in the 2D model, indicating that the double-layer coil can be studied as two coils. The distances of two adjacent turns in the interlaced double-layer coil are *D*_0_ and *D*_1_, and the distances of two adjacent turns in the parallel double-layer coil are *Z*_0_, *D*_0_, and *D*_1_. The magnetic field strength |***H***(*i*)| can be solved in accordance with Eqs. (4)– (11).
  The calculation results of AC resistance *R*_ac_ with single-layer and double-layer coils are shown in Fig. 12 (*R*_in_ = 0.1 m, *R*_out_ = 0.15 m, *N* = 20, *r*_0_ = 1.18 mm).

**Figure 12:**
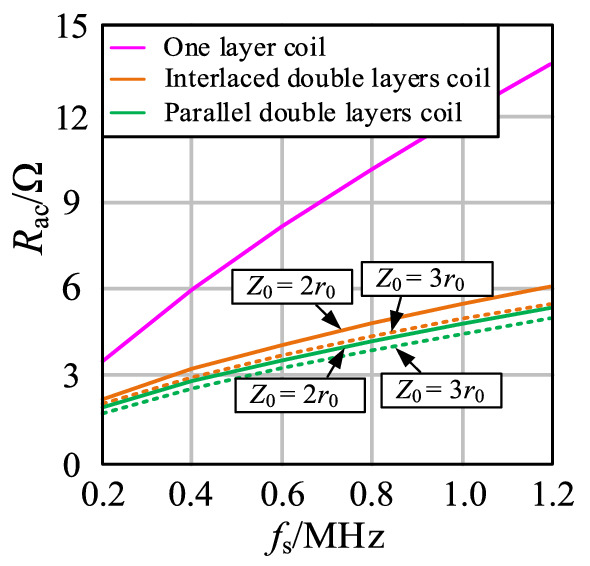
Comparison of *R*_ac_ between single-layer coil and double-layer coil.

In accordance with Eqs. (12), (13), and (14), the turn spacings of coils are as follows:

1. The turn spacing of the single-layer coil is *d*_0_ = 0.272 mm.
2. When the moving distance *Z*_0_ = 2*r*_0_, the turn spacings of the interlaced double-layer coil are *D*_0_ = 1.175 mm and *D*_1_ = 2.904 mm. The turn spacings of parallel double-layer coil are *D*_0_ = 2.983 mm and *D*_1_ = 2.903 mm.
3. When the moving distance *Z*_0_ = 3*r*_0_, the turn spacings of the interlaced double-layer coil are *D*_0_ = 2.051 mm and *D*_1_ = 2.904 mm. The turn spacings of parallel double-layer coil are *D*_0_ = 3.983 mm and *D*_1_ = 2.903 mm.

As shown in Fig. 12, the double-layer coil can effectively reduce *R*_ac_, and the optimization effect is more obvious with the increase in frequency and *Z*_0_. The moving distance *Z*_0_ is restricted by coil volume and transmission distance. Thus, the moving distance cannot be extremely large. Coil with three or multiple layers can be used. However, the thickness of the coil increases with the increase in the number of coil layers.

### Simulation and experimental verification

**Figure 13:**
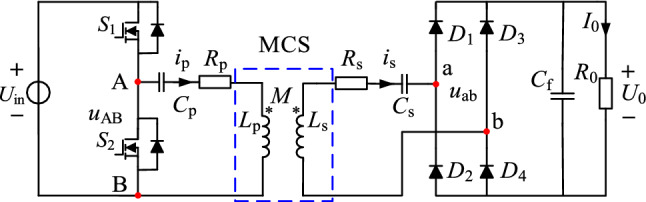
Circuit model of S/S compensation topology.

The circuit model of the S/S compensation topology WPT system is shown in Fig. 13.

*U*_in_ is the DC voltage source, *M* is the mutual inductance, *C*_f_ is the filter capacitor, and *R*_0_ is the load resistance. *L*_p_, *R*_p_, *C*_p_, and *i*_p_ are the transmitter coil's self-inductance, AC resistance, compensation capacitor, and currents. *L*_s_, *R*_s_, *C*_s_, and *i*_s_ are the receiver coil's self-inductance, AC resistance, compensation capacitor, and currents.

The MCS is shown in Fig. 13. The parameters of the coil are shown in Table 1.

**Table 1:** The parameters of the coil.

Variables	Value
Coil size	*R*_in_ = 0.1 m, *R*_out_ = 0.15 m
Wire gauge	*r*_p_ = 0.6 mm, *r*_s_ = 1.18 mm
Turns	*N*_p_ = 40, *N*_s_ = 20
Transmission distance	*h* = 0.1 m
Turn spacing	Uniform

Three different schemes are adopted for comparison.

1. Comparison case.

The 2D model of the coil is shown in Fig. 1.

(2) Optimal case 1.

The coils are designed as interlaced double-layer coils, and the moving distances of the transmitter and receiver coil are 1.2 and 2.36 mm, respectively.

(3) Optimal case 2.

The coils are designed as parallel double-layer coils, and the moving distances of the transmitter and receiver coil are 1.2 and 2.36 mm, respectively.

Tables 2 and 3 compare the simulation and measured inductance of coils in three schemes at fs = 200 kHz. The measured coil parameters are obtained by the impedance analyzer WK6500B and the simulation coil parameters are obtained by FEA simulation software.

**Table 2:** Inductance of the three schemes in the simulation.

Scheme	*L*_p_/μH	*L*_s_/μH	*M*/μH
Comparison case	611.31	148.04	64.19
Optimal case 1	607.21	146.63	64.05
Optimal case 2	602.42	144.56	64.03

**Table 3:** Inductance of the three schemes in the experiment.

Scheme	*L*_p_/μH	*L*_s_/μH	*M*/μH
Comparison case	608.76	150.06	64.20
Optimal case 1	605.34	148.73	64.11
Optimal case 2	604.12	148.67	64.05

As shown in Tables 2 and 3, the inductance values of the three schemes are consistent, and the measured inductance values are the same as the simulation results.

When the coil sizes (*R*_in_ = 0.1 m, *R*_out_ = 0.15 m) are the same in different schemes, the mutual inductance *M* of MCS coils is only related to the product of *N*_p_ and *N*_s_. So, the mutual inductance *M* of MCS coils is the same in the three schemes. The consistency of the simulation and measured mutual inductance *M* values in the three schemes are excellent, and the maximum error between the measured *M* values is below 0.3% in accordance with Tables 2 and 3. Thus, the three schemes can obtain the same power when the input voltage *U*_in_ and load resistance *R*_0_ are the same.

**Figure 14:**
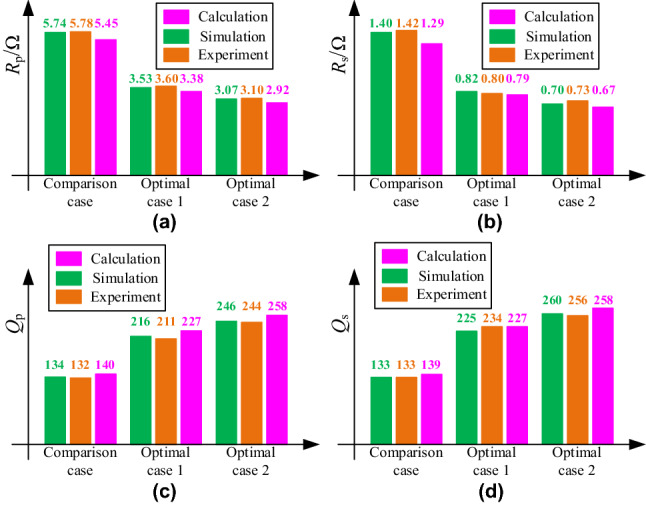
The AC resistance and quality factor of coils in three schemes: **a** *R*_p_; **b** *R*_s_; **c** *Q*_p_; **d** *Q*_s_.

The AC resistance and quality factor of coils in three schemes at *f*_s_ = 200 kHz, as shown in Fig. 14.

In Fig. 14, the simulation value of coil resistance is mostly the same as the measured value. The error between the calculated value obtained using the proposed calculation method and the simulation value is less than 5%. Compared with the comparison scheme, the double-layer coil scheme can greatly reduce the AC resistance and improve the quality factor *Q* of coils.

An error is found between the calculation results and the simulation values. This condition is because when the distance of the turn spacing is small, errors may occur in the calculation of current position.

**Figure 15:**
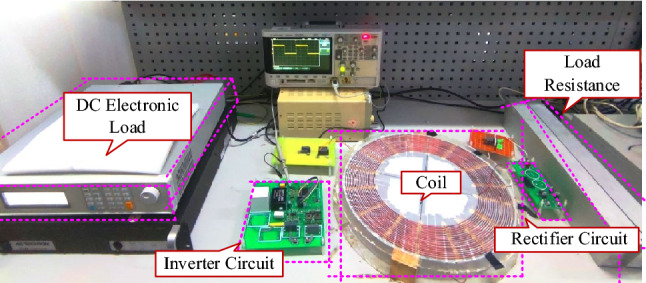
Experimental platform.

The experimental platform is built, as shown in Fig. 15, where *U*_in_ = 250 V, *R*_0_ = 50 Ω, *P*_0_ = 72 W, and *f*_s_ = 200 kHz. The compensation capacitance parameters are shown in Table 4.

**Table 4:** Compensation capacitance parameters.

Scheme	*C*_p_/nF	*C*_s_/nF
Comparison case	1.03	4.20
Optimal case 1	1.05	4.28
Optimal case 2	1.05	4.28

**Figure 16:**
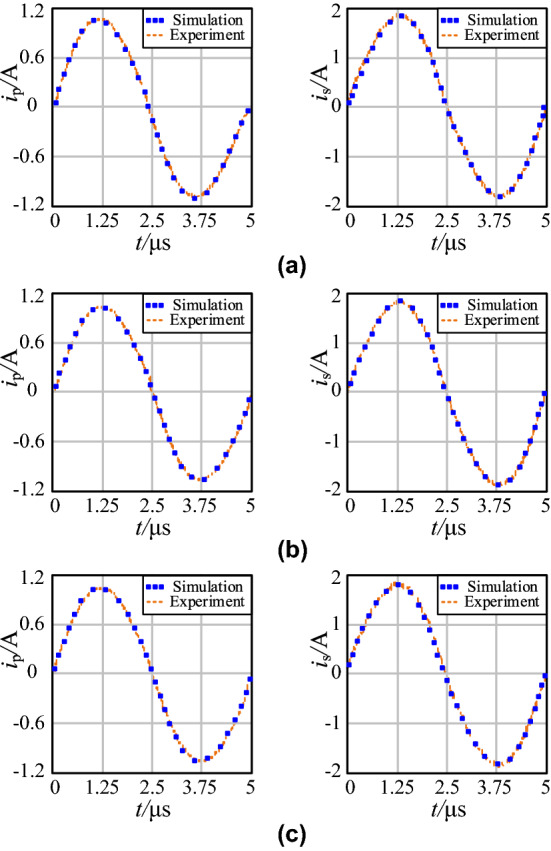
Waveforms of *i*_p_ and *i*_s_ in the three schemes: **a** comparison case; **b** optimal case 1; **c** optimal case 2.

The simulation and measured current waveforms of the transmitter and receiver coil in three schemes are shown in Fig. 16, where *i*_p_ is transmitter coil current and *i*_s_ is receiver coil current.

It can be seen that regardless of *i*_p_ or *i*_s_, the measured and simulation current waveforms are almost the same.

**Figure 17:**
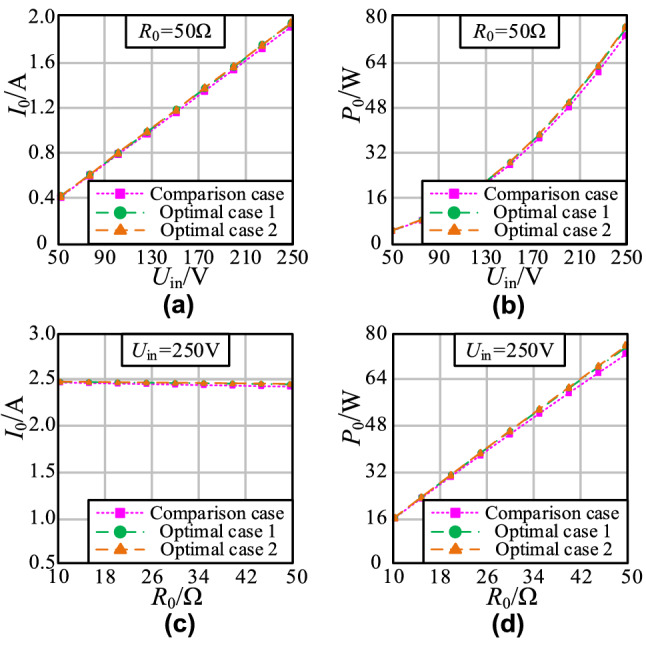
The curve of output current and output power: **a** *I*_0_-*U*_in_; **b** *P*_0_-*U*_in_; **c** *I*_0_-*R*_0_; **d** *P*_0_-*R*_0_.

The measured waveforms of output current *I*_0_ and output power *P*_0_ in three schemes are shown in Fig. 17.

As shown in Figs. 17a and b, the *I*_0_ and *P*_0_ values of the three schemes are approximately equal when the load resistance *R*_0_ is rated load, which increases with an increase in the input voltage *U*_in_. The *I*_0_ and *P*_0_ values of the three schemes are the same at different *R*_0_, as shown in Fig. 17c,d.

**Figure 18:**
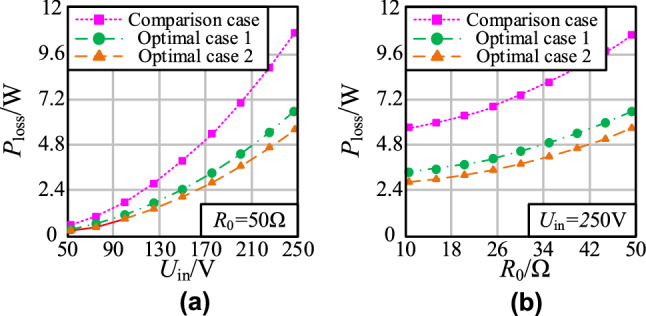
The loss curve of MCS coils: **a** *P*_loss_-*U*_in_; **b** *P*_loss_-*R*_0_.

In accordance with the AC resistance and quality factor of coils in Fig. 14, the total loss of MCS coils can be obtained by *P*_loss_ = *I*_p_^2^*R*_p_ + *I*_s_^2^*R*_s_, as shown in Fig. 18. The double-layer coil scheme can greatly reduce the loss of MCS. At the rated operating condition, the coil loss of MCS is reduced by more than 40%.

**Figure 19:**
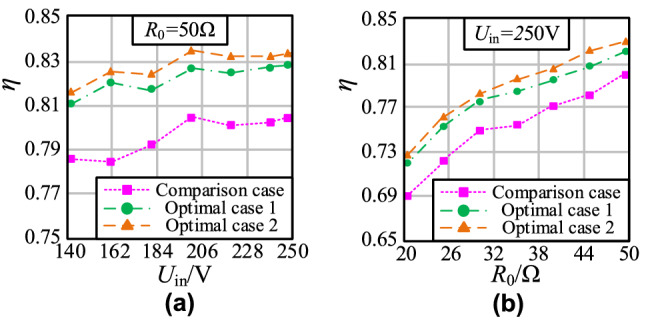
Efficiency curves: **a** *η*-*U*_in_; **b** *η*-*R*_0_.

The efficiency is tested by the YOKOGAWA PX8000 power oscillograph (sampling accuracy: 12 bit, test bandwidth: DC-20 MHz) to analyze the optimization effect of the double-layer coil on the WPT system. Furthermore, the efficiency curves at different input voltage and load resistance are shown in Fig. 19.

The curve in Fig. 19a shows that the WPT system efficiency *η* is always higher than the comparison case with *U*_in_ increases when MCS coils use double-layer coils. It can be seen in Fig. 19b that the optimization effect of the parallel double-layer coil is the best when the system is full load. At this time, using the double-layer coil to optimize MCS coils can improve the efficiency by 4.3% compared with the traditional scheme. The double-layer coil structure optimization scheme is valid for enhancing the efficiency of the WPT system.

## Conclusion

This study proposes the optimized double-layer coil structure for the solid planar spiral coil. The conclusions are summarized as follows:

- This paper analyzes the AC resistance of the single-turn coil, then deduces the planar spiral coil's AC resistance model and proposes the coil's AC resistance model, which can take the mutual influence between the transmitter and the receiver coil into consideration.
- The affecting factors are analyzed based on the loss model. The proximity effect is obvious with the decrease in turn spacing, and the coil loss increases. The AC resistance of the coil can be reduced by optimizing the turn spacing.
- A new scheme to optimize MCS coils is proposed-a double-layer coil structure. This scheme can greatly reduce the coil AC resistance and improve the quality factor without changing the coil turns, wire gauge, and footprint.
- Compared with the air planar spiral coil with a one-layer structure, the parallel double-layer coils can reduce the AC resistance of the coil by more than 40% and the system efficiency of optimized MCS coils is increased by 4.3%.

## Data Availability

The authors declare that the data supporting the findings of this study are available within the article and its supplementary information files. All other relevant data are available from the corresponding author upon reasonable request.
